# Structure of a Highly Conserved Domain of Rock1 Required for Shroom-Mediated Regulation of Cell Morphology

**DOI:** 10.1371/journal.pone.0081075

**Published:** 2013-12-09

**Authors:** Swarna Mohan, Debamitra Das, Robert J. Bauer, Annie Heroux, Jenna K. Zalewski, Simone Heber, Atinuke M. Dosunmu-Ogunbi, Michael A. Trakselis, Jeffrey D. Hildebrand, Andrew P. VanDemark

**Affiliations:** 1 Department of Biological Sciences, University of Pittsburgh, Pittsburgh, Pennsylvania, United States of America; 2 Department of Chemistry, University of Pittsburgh, Pittsburgh, Pennsylvania, United States of America; 3 Department of Biology, Brookhaven National Laboratory, Upton, New York, United States of America; University of North Carolina at Chapel Hill, United States of America

## Abstract

Rho-associated coiled coil containing protein kinase (Rho-kinase or Rock) is a well-defined determinant of actin organization and dynamics in most animal cells characterized to date. One of the primary effectors of Rock is non-muscle myosin II. Activation of Rock results in increased contractility of myosin II and subsequent changes in actin architecture and cell morphology. The regulation of Rock is thought to occur via autoinhibition of the kinase domain via intramolecular interactions between the N-terminus and the C-terminus of the kinase. This autoinhibited state can be relieved via proteolytic cleavage, binding of lipids to a Pleckstrin Homology domain near the C-terminus, or binding of GTP-bound RhoA to the central coiled-coil region of Rock. Recent work has identified the Shroom family of proteins as an additional regulator of Rock either at the level of cellular distribution or catalytic activity or both. The Shroom-Rock complex is conserved in most animals and is essential for the formation of the neural tube, eye, and gut in vertebrates. To address the mechanism by which Shroom and Rock interact, we have solved the structure of the coiled-coil region of Rock that binds to Shroom proteins. Consistent with other observations, the Shroom binding domain is a parallel coiled-coil dimer. Using biochemical approaches, we have identified a large patch of residues that contribute to Shrm binding. Their orientation suggests that there may be two independent Shrm binding sites on opposing faces of the coiled-coil region of Rock. Finally, we show that the binding surface is essential for Rock colocalization with Shroom and for Shroom-mediated changes in cell morphology.

## Introduction

Coordinated cellular processes that alter cell and tissue morphology, such as apical constriction, are often driven by the assembly and activation of contractile networks of F-actin and non-muscle myosin II (reviewed in [1]). This contractility and the resulting changes in cell shape are required for the proper development of numerous tissues including the vasculature, heart, central nervous system, kidney, and gut [2]–[14]. The signaling and mechanistic aspects of apical constriction have been widely studied and have recently been placed in a cellular framework. It has been shown in several cellular and genetic model systems that apical constriction is driven largely by the pulsatile contraction of a cortical mesh of actin bundles that are indirectly linked to the apically positioned cell-cell adhesions mediated by the cadherins. The mechanical force for contraction is supplied by motor activity of myosin II (reviewed in [15]). The trigger for apical constriction comes via signaling pathways that result in the phosphorylation of myosin regulatory light chain (MRLC) at serine19, which is correlated with actin-stimulated ATPase activity, suggesting this modification is driving changes in cytoskeletal architecture [16], [17]. MRLC phosphorylation at serine 19 has been reported for several kinases including Calcium/Calmodulin dependent protein kinase [18], Protease activated protein kinase I [19], and Rho-associated kinase (Rock) [20], suggesting that myosin contractility and cytoskeletal dynamics are cellular characteristics that are regulated by a wide range of environmental cues.

Vertebrates have two highly related Rock proteins, Rock1 and Rock2, which share 65% sequence identity to each other. Both contain an N-terminal kinase domain, a centrally located coiled coil region and C-terminal pleckstrin homology (PH) and cysteine-rich domains. The Rock kinase domain has a typical Ser/Thr kinase fold, similar to protein kinase A, consisting of two kinase lobes linked by a hinge [21]. N-terminal and C-terminal extensions from the kinase domain facilitate dimerization and are also required for activity [22], [23]. The kinase domains dimerize in a head-to-head arrangement with active sites located along a single face of the dimer and positioning the adjacent sequences for coiled-coil formation [22].

**Figure 1:**
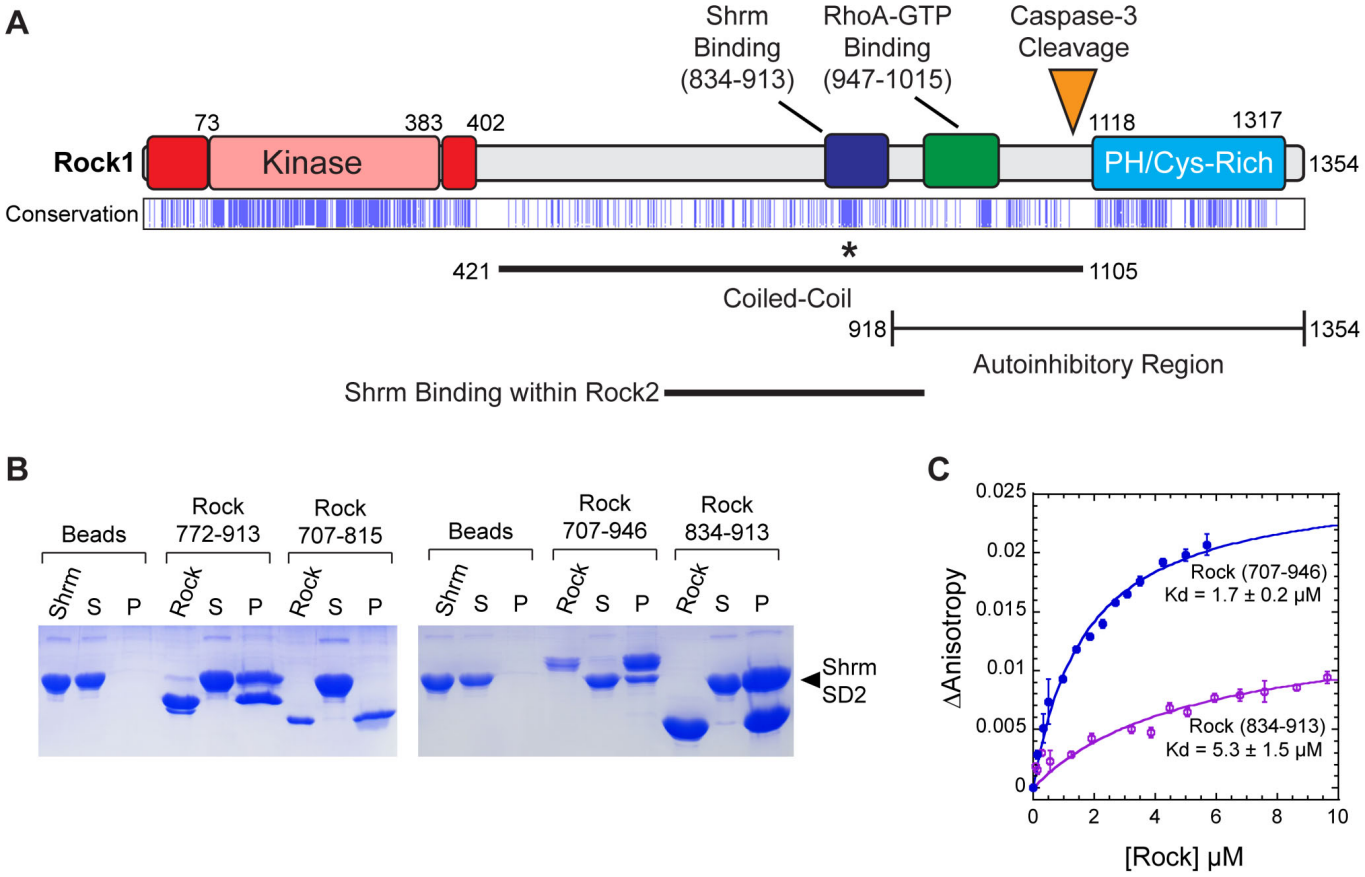
A Central region within the coiled-coil domain interacts with Shrm SD2. A) Diagram of Rock1 domain structure. Domains and their boundaries within Rock1 are indicated. N- and C-terminal extensions on the Rock1 kinase domain are shown in red. Sequence conservation from a multiple sequence alignment of 22 Rock sequences is shown with sequence positions containing 90% identity indicated in blue. B) Identification of a minimal Shrm SD2 binding domain within Rock1. Purified untagged human Shrm2 SD2 was mixed with beads pre-bound to the indicated his-tagged fragment of Rock1. Complexes were precipitated by spinning down the beads and the resulting samples were resolved on SDS-PAGE. P, pelleted beads; S supernatant. C) Rock fragments were assayed for binding to Shrm SD2. Increasing concentrations of Rock1 (707–946) or (834–913) were added to a reaction mixture containing 50 nM Oregon-Green labeled human Shrm2 SD2 domain in a fluorescence spectrophotometer. The binding isotherm was fit to Equation 1 using a non-linear regression to determine binding affinity (*K_d_*).

Rock catalytic activity is inhibited by a direct intramolecular interaction between the kinase domain and sequences within a large C-terminal fragment of Rock containing 200 residues of the coiled-coil region, the PH domain , and the cysteine-rich domain [24] (Figure 1A). Relief of Rock autoinhibition can be achieved through several independent mechanisms involving interaction or modification with this autoinhibitory region. These include lipid binding to the PH domain [25], [26], removal of the PH domain via proteolytic cleavage by Caspase-3 [27], [28], or the binding of other proteins such as Dynamin I [29] or the small GTPase RhoA [30]. Of these, the interaction with RhoA is probably the most intensely studied and widely utilized at the cell and organismal level. RhoA recognizes binding sites within the coiled-coil region of Rock in a GTP dependent manner [30] using canonical Switch I and II loops [31]. Structural studies have shown that the Rho-binding domain (RBD) is maintained as a parallel dimeric coiled-coil after RhoA binding with the RhoA interface composed of residues from both Rock proteins [31], [32]. Other RhoA interacting proteins, such as Protein-kinase N, use a single chain antiparallel coiled-coil to bind a separate surface on RhoA, thus highlighting the mechanistic diversity this class of effector proteins can use to modulate function [33].

The Shroom (Shrm) family of actin-binding proteins play essential roles in the development of multiple tissues, including the nervous system, eyes, heart, vasculature, and gut, by mediating the formation of contractile actomyosin networks that guide changes in cell shape and migration [3], [8]–[10], [14], [34]–[40]. Of the four vertebrate family members, Shrm3 is the most extensively characterized and serves as a model for the function of other Shrm proteins. Using both animal models and in vitro cell culture systems, Shrm3 has been shown to elicit apical constriction, a behavior of epithelial cells that results in decreased apical area and is thought to facilitate developmental processes such as tissue invagination and bending [8], [36], [37], [41]. To accomplish this task, Shrm3 is localized to apically positioned sites of cell-cell adhesion in polarized cells, where it directly interacts with Rock through a highly conserved domain on its C-terminus called the Shrm Domain 2 (SD2). The interaction between the SD2 and Rock is essential for Shrm3-induced apical constriction. Although several Rock effectors are linked to regulation of the cytoskeleton, previous studies indicate that Rock elicits apical constriction through the activation of non-muscle myosin II, as inhibition of Rock or Myosin II has been shown to prevent Shrm3-induced apical constriction [36]. The SD2 domain is an unusual three-segmented antiparallel coiled-coil that contains no sequence or structural homology to other Rock activators [42], and thus it is unclear how SD2 binding regulates Rock kinase activity. Shrm3 may function to localize Rock to the apical surface where it is then activated by another signal or, alternatively, Shrm3 may function to both recruit and activate Rock. In either scenario, the Shrm-Rock interaction results in the assembly of a contractile actomyosin network in the zonula adherens and subsequent constriction of the apical domain of the cell. Previous studies indicate all Shrm family proteins may function in an analogous manner and that the Shrm-Rock complex is conserved in most animals [34], [35], [43]–[45]. However, not all Shrm proteins exhibit the same subcellular distribution, suggesting that an important aspect of Shrm activity may be to initiate myosin II activity in different regions of the cell in order to elicit different cellular behaviors [44].

In order to gain insight into the mechanism of Shrm-mediated activation of Rock, we have identified a minimal stable domain within the coiled-coil domain of Rock that facilitates Shrm binding. We have determined the structure of this domain using x-ray crystallography, revealing a dimeric, parallel coiled-coil with conserved surfaces that mediate binding to Shrm and subcellular colocalization, as well as Shrm-mediated reorganization of the cytoskeleton and changes in cell shape. These data indicate that Shrm and Rock comprise a conserved signaling module required for changes in cell architecture and tissue morphology.

## Materials and Methods

### Protein expression and purification

Coding sequences for all Rock1 SBD constructs were amplified by PCR and cloned into the bacterial expression vector pET151-D/Topo (Invitrogen), which directs expression of an N-terminal His_6_ tag that can be removed by digestion with TEV protease. Protein expression was performed in the bacterial strain BL21(DE3) codon plus (RIPL) using ZY autoinduction media at room temperature [46] for ∼24 hours. Cells were lysed via homogenization in 25 mM Tris pH8.0, 500 mM NaCl, 10% glycerol, 5 mM Imidazole, 5 mM beta-mercaptoethanol. The lysate was cleared by centrifugation at 100,000× *g* prior to chromatography. Wild-type and SER mutant Rock1 SBD proteins were purified by nickel affinity chromatography, followed by overnight digestion with TEV protease. A second round of nickel affinity purification was performed to remove the liberated His-tag and TEV protease, followed by anion exchange chromatography and gel filtration using a Sephacryl S-200 (GE Healthcare). Peak fractions were pooled and concentrated. Wild-type and mutant Rock1 SBD were concentrated to 15 mg/ml prior to crystallization in 150 mM NaCl, 20 mM HEPES pH 7.5 and 1 mM β-mercaptoethanol using a Vivaspin concentrator (Millipore) prior to crystallization. The purity was typically >99% as verified by SDS-PAGE. Selenomethionine substituted mutant Rock1 SBD was expressed using PASM media, and purification was essentially the same as the native protein [46]. Human Shrm2 SD2 domain (1427–1610) was cloned into the pET151/D-TOPO vector and expressed in BL21(DE3) Codon+(RILP) using ZY autoinduction media. The purification was similar as described for Drosophila Shrm SD2 domain [42].

### Mutagenesis of human Rock1 SBD proteins

The surface entropy reduction mutation and all of the mutations in hRock1 SBD (707–946 and 834–913) were made using the QuikChange Site-Directed Mutagenesis kit (Stratagene). The mutant Rock proteins were expressed and purified in a manner similar to the wild-type proteins.

### Crystallization and Structure Determination

Crystals of Rock1 SBD (834–913) containing the SER mutant were grown using the vapor diffusion technique against a reservoir solution containing 0.1 M Citrate pH 6.0, and 1.0 M ammonium sulfate. Crystals were optimized by seeding at 4°C and grow to 300×100×50 µm over the course of two weeks. Crystals were cryoprotected by transition into a buffer containing 0.1 M citrate pH 6.0, 2.5 M ammonium sulfate, 0.15 M NaCl, and 20% glycerol and flash frozen under liquid nitrogen prior to data collection. The same procedure was used to crystallize and cryoprotect selenomethionine (SeMet) substituted crystals.

Diffraction data from all crystals was collected at beamline X25 at the National Synchrotron Light Source. Integration, scaling, and merging of diffraction data was performed using HKL2000 [47]. Crystals of Rock1 SBD^SER^ belong to space group C2_1_ with *a* = 142.5 Å, *b* = 56.2 Å, *c* = 80.7 Å, and β = 119.1°, and are highly anisotropic with diffraction extending to 2.5 Å resolution in the a* and c* direction but limited to 3.1 Å in the b* direction. Initial phases were obtained from crystals of selenomethionine substituted protein via the SAD method at 4.0 Å using SHELX C/D/E which found all four selenium sites [48]. Data from these crystals were also highly anisotropic and the map quality was only sufficient to build an initial model using Coot [49]. This model was further refined against the native dataset after ellipsoidal truncation using the diffraction anisotropy server [50]. The model was improved using a combination of simulated annealing, rigid body, positional, B-factor, and TLS refinement within Phenix [51]. Model quality was monitored using MolProbity [52].

### Complex Formation

Wild-type or mutant Rock1 707–946 (10 µM) was mixed with human Shrm2 SD2 domain (also at 10 µM) and incubated at room temperature for 20 minutes. Shrm-Rock complexes were resolved on 12% native PAGE gels run at 4°C and stained with Coomassie Blue.

### Fluorescent Labeling of Human Shroom2 SD2

Recombinant human Shrm2 SD2 domain (1427–1610) was N-terminally labeled with Oregon Green 488 Succinimidyl ester (Invitrogen) in amino labeling buffer (20 mM Hepes pH 7.4, 100 mM NaCl, 5% Glycerol) in a manner similar to [53]. Labeling reactions included 10× molar excess of fluorophore at room temperature for 2 h. Excess fluorophore was removed from the samples through extensive dialysis with labeling buffer. The labeling efficiency was quantified using the extinction coefficient of the dye compared with the protein concentration determined from a standard curve using a Bradford assay and found to be essentially 1∶1.

### Fluorescence Anisotropy Binding Experiments

Fluorescence anisotropy measurements were performed in (20 mM Hepes pH 7.4, 100 mM NaCl, 5% Glycerol) using 20 nM of N-terminally labeled Shrm2 SD2-Oregon Green 488 and increasing concentrations of Rock1 SBD. Measurements were collected as described previously using a Floromax-3 fluorimeter (Horiba Jobin Yvon) [54]. Labeled proteins were excited at 496 nm and emission was monitored at 524 nm using 5-second integration times for three consecutive readings. The reported anisotropy values (r) are the average of at least three independent experiments and fit by a non-linear least squares analysis using Kaleidagraph (Synergy, Reading, PA) to a single binding model: *r* = (ΔA×[P])/(*K_d_*+[P]) (Equation 1) where *A* is the amplitude, P is the concentration of titrated Rock1 SBD, and *K_d_* is the dissociation constant.

### Shrm-Rock Pulldowns

*In vitro* Pull-down assays were performed using the indicated His-tagged fragments of human Rock1 and untagged human Shrm2 SD2 (1427–1610). 25 µg of purified His-tagged Rock protein was immobilized to 50 µl of nickel beads (Qiagen), and washed three times with binding buffer (2% glycerol, 100 mM NaCl, 7 mM Imidazole, 1 mM β-mercaptoethanol, and 20 mM Tris pH 8.0) to remove unbound protein. A 2× molar excess of untagged Shrm was then incubated with Rock conjugated beads in binding buffer for 10 minutes at room temperature. Beads were then spun down and a supernatant sample taken prior to three washes with binding buffer. The beads were then incubated with 4× SDS-Loading buffer and resolved on 15% SDS-PAGE and detected by Coomassie staining. Bead control sample was run by substituting binding buffer for the His-tagged Rock protein. For pulldowns from cell lysates, 293 cells were transiently transfected with the indicated Rock-SBD expression plasmids and grown overnight. Cells were lysed in RIPA buffer and cleared by centrifugation. Equal amounts of GST or GST-mShroom3 SD2 bound to glutathione sepharose were added to the lysate and incubated at 4°C for 2 hours with rocking. Beads were washed 3 times with RIPA buffer and resuspended in SDS-PAGE sample buffer. Total cell lysate and eluted proteins were resolved by SDS-PAGE, transferred, and detected using mAb 9E10 and SuperSignal West Pico Chemiluminescent Substrate (Thermo). Luminescence was detected using Fujifilm LAS-3000 imager. 293HEK cells were used due to higher transfection efficiency.

### Chemical Crosslinking

Wild-type and SBD^SER^ were incubated for 25 minutes with 0.002% glutaraldehyde in a reaction buffer containing 25 mM HEPES pH 7.5, 8% Glycerol, 500 mM NaCl and 5 mM β-ME, at a final protein concentration of 26 µM. The crosslinking reaction was stopped with the addition of Tris pH 8.0 to a final concentration of 0.2 M. Crosslinked species were then visualized by SDS-PAGE with Coomassie blue staining.

### Immunofluorescence and siRNA mediated knock-down studies

Cos7 or T23 MDCK cells were grown in DMEM/10% FBS/L-glut/pen-strep or EMEM/10% FBS/L-glut/pen-strep. Cells were transfected with indicated expression vectors using Lipofectamine. Briefly, cells were trypsinized and mixed with DNA/Lipofectamine complexes and plated onto fibronectin-coated cover slides or transwell filters for 24 hours. Cells were fixed with 4% paraformaldehyde and stained to detect Shroom3 (UPT132, [36]), or myc-tagged hRockI 681–942. Primary antibodies were detected using Alexa-conjugated secondary antibodies. For Rock knock-down studies, siRNAs specific for canine Rock1 and Rock2 mRNA were designed using the Dharmacon design tools based gene accession numbers XM_537305 and XM_540083. Three siRNAs specific for Rock1 (1.1, GAAAUAGCAAGAGAACUAUU; 1.2, GAGAAUUGAAAGAGAGAAAUU; and 1.3, GCGAAAUGGUGUAGAAGAAUU) and Rock2 (2.1, UGAAAGAAAUGGAGAAGAAUU; 2.2, CGAACAAGAUAAAGAACAAUU; and 2.3, UGAAGAAAGUCAAGAGAUUUU) were tested for efficient knock-down via western blotting using rabbit anti-Rock antibodies (Bethyl). Briefly, parental T23 MDCK cells were transfected with individual siRNAs (10 µl of 20 µM siRNAs in a final volume of 2 ml) and cells were grown for 3 days prior to lysis and blotting. siRNAs 1.2 and 2.1 were used for all subsequent experiments. T23 MDCK+EndoShrm3 cells (5×10^5^) were transfected with 5 µL of Lipofectamine 2000 and 10 µL (20 µM) each siRNA in a final volume of 2 mL in a 35 mm plate. After two days, doxycycline was withdrawn to induce the expression of Endo-Shrm3. After 48 hours of siRNA transfection, cells were transiently transfected with expression plasmids for Rock1 variants using Lipofectamine as previously described [36]. Cells were plated onto transwell membranes and allowed to grow overnight. Membranes were then stained to detect Rock1 and ZO1. Images were captured using either an Olympus Flo-View or Bio-Rad Radiance confocal microscope. Images were processed using either Photoshop or ImageJ. For quantifying rescue of apical constriction by the various Rock proteins, the apical areas of only Rock-expressing cells was measured using ImageJ. The apical area was considered to be that region of the cell encircled by ZO1 staining. Statistical significance was determined using Students t-test . This experiment was repeated in three separate, independent trials.

### Accession Codes

Coordinates and structure factors for Rock1 SBD^SER^ have been deposited at the Protein Data Bank and assigned the identifier 4L2W.

## Results

### Identification of a minimal Shrm binding domain

Multiple lines of investigation, including biochemical, structural, and cellular analysis, have characterized and demonstrated the importance of the Shrm-Rock interaction in the regulation of cytoskeletal organization, cell shape, and tissue morphogenesis [5], [8]–[10], [37], [42]. Within Shrm, this interaction is mediated by a highly conserved Shrm-domain 2 (SD2) found at the C-termini of all Shrm proteins identified to date [35], [37], [42], [44]. Shrm binding capacity had been demonstrated for a large, central region within the coiled-coil domain of Rock2 (amino acids 698–947) that is distinct from the RhoA-binding domain, however residues that specify this interaction within Rock1 have not been identified [37]. Therefore, we sought to define the Shrm-binding region within Rock and determine the mechanism by which it interacts with the SD2.

Since both Rock1 and Rock2 have been shown to bind the SD2 motif of Shrm, we reasoned there is a conserved sequence motif within Rock that would mediate this interaction. Mapping sequence conservation within an alignment of 22 Rock protein sequences, we noted a region of high conservation within the SD2 binding region (Figure 1A, asterisk). Disorder profiles of the Rock1 sequence predict 834–913 as a stably folded fragment containing this region of conservation. We then tested Rock1 proteins containing residues 707–815, 707–913, 772–913, or 834–913, for the ability to support Shrm SD2 binding in a pull-down assay. In this assay, His_6_-tagged Rock1 fragments containing residues 707–913, 772–913, or 834–913 bound to nickel resin were sufficient to pull-down untagged human Shrm2 SD2 (Figure 1B). In contrast, the 707–815 fragment of Rock1 was unable to pull down the SD2, suggesting that the necessary sequence for Shrm binding contains amino acids 815–913. We examined this interaction quantitatively by labeling human Shrm2 SD2 with Oregon-Green at its N-terminus and monitoring fluorescence anisotropy throughout a titration of Rock (Figure 1C). We tested the ability of Rock1 proteins to bind in this assay; the first, containing residues 707–946 corresponds closely to the fragment initially shown to mediate Shrm binding [37], while the second, comprised of residues 834–913, was identified based on conservation and verified in our pulldowns. While binding affinity was somewhat reduced in this assay for the smaller 834–913 fragment, both Rock1 proteins were still capable of binding SD2 with affinities comparable to that observed between Drosophila Rock and Shrm [42]. Together, these data suggest that there is a stable Shrm binding domain (SBD) composed of residues 834–913 of hRock1.

### The structure of Rock1 SBD

It is interesting to note that the Rock SBD does not overlap with those sequences previously identified as being involved in Rock autoinhibition, raising the possibility that the mechanism by which Shrm relieves Rock autoinhibition may be distinct from the canonical activator RhoA. Alternatively, Shrm binding to Rock may prevent autoinhibition in a manner that is similar to RhoA but is functionally independent from RhoA. To understand the details of the Rock-Shrm interaction and their mechanistic implications, we obtained crystals of the human Rock1 SBD. However, these crystals exhibited low resolution and anisotropic diffraction and were unsuitable for structure determination. Diffraction quality was greatly improved through the introduction of a triple alanine mutation, ^884^EKE^886^, suggested by the Surface Entropy Reduction server [55] and termed SBD^SER^.

**Figure 2:**
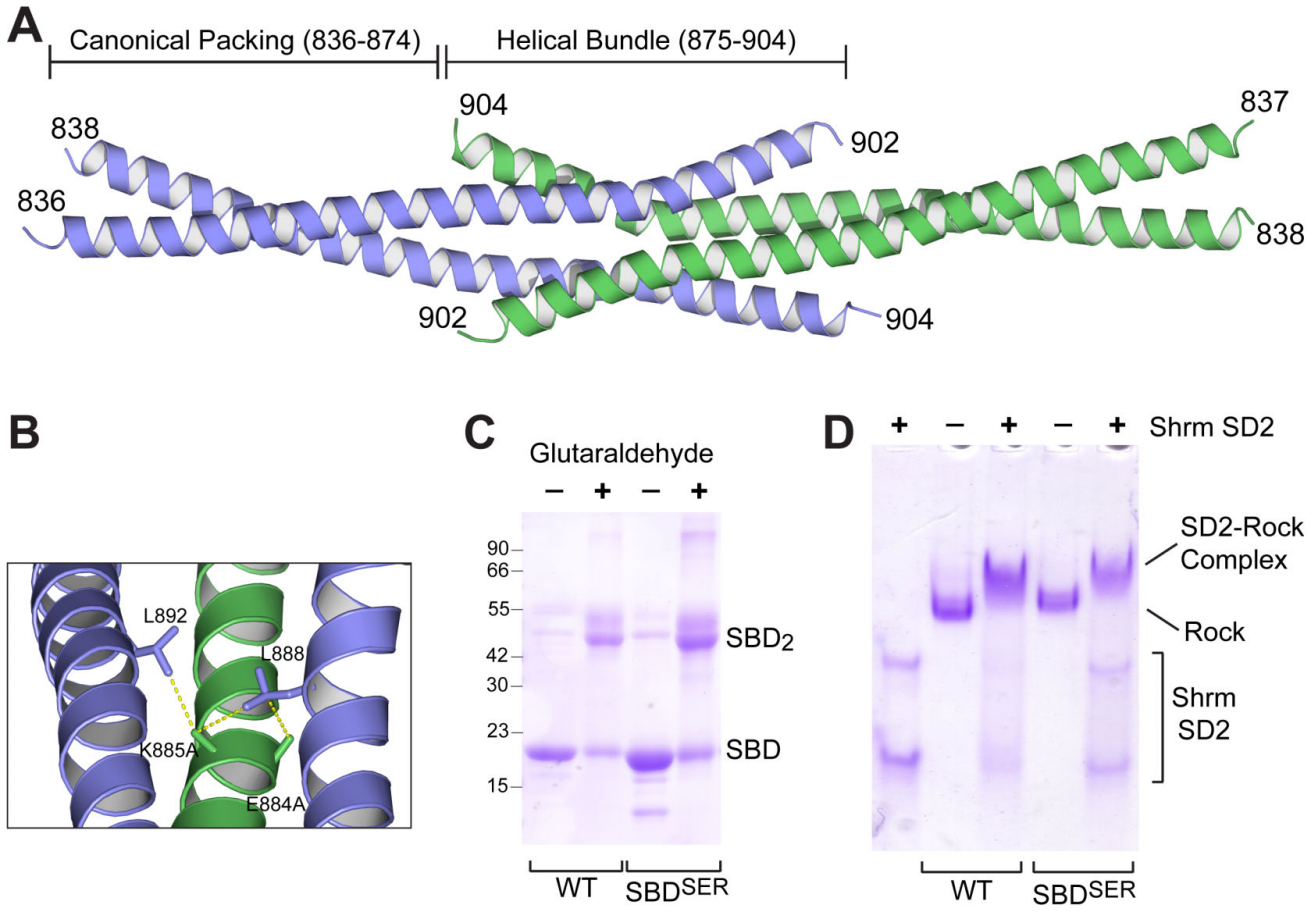
Structure of Rock1 834–913. A) Two dimers of Rock1 SBD (blue and green) pack within the crystal in a tail-to-tail arrangement, forming a central helical bundle flanked by regions of canonical coiled-coil. B) Cartoon view of interactions within the helical bundle mediated by the residues 884–886. Hydrophobic interactions mediated by A884 and A885 with canonical interface residues L888 and L892 are highlighted. C) SBD^SER^ does not alter the oligomeric state of SBD in solution. WT SBD and SBD^SER^ were crosslinked with 0.002% glutaraldehyde and the resulting species separated by SDS-PAGE. D) Rock1 SBD containing the SER mutant can still bind Shrm SD2. Human Shrm2 SD2 domain was incubated with the indicated Rock1 fragments and resolved by native gel electrophoresis.

Crystals of SBD^SER^ belong to space group C2_1_ and also exhibit anisotropy with diffraction extending to 2.5 Å along a* and c* but only 3.1 Å along the b* plane. The diffraction data were then filtered to retain data within these resolution limits [50], [56] (see Materials and Methods and Table 1 for additional details regarding the structure determination process). Phases were determined using the SAD method, and the final model was refined against native data at 2.5 Å resolution to R_work_ and R_free_ values of 23.6% and 27.6% respectively. SBD^SER^ crystals contain two parallel coiled-coil dimers in the asymmetric unit packed in a tail-to-tail arrangement (Figure 2A). Each SBD monomer is an entirely helical segment ∼100 Å in length and the completed model minimally contains residues 838–902 from each molecule. The two dimers in the asymmetric unit are essentially equivalent with an r.m.s.d. of 1.0 Å over 131 Cα atoms.

**Table 1:** Crystallographic Data collection and refinement statistics for human Rock1 SBD^SER^.

	SeMet	Native
**Data Collection**		
Space Group	C2_1_	C2_1_
Cell Dimensions		
*a* (Å)	141.6	142.5
*b* (Å)	56.1	56.2
*c* (Å)	80.4	80.7
α, β, γ (°)	90, 119.0, 90	90, 119.1, 90
Resolution (Å)	50.0-2.4 (2.44-2.40)	50.0-2.5 (2.54-2.50)
Unique Reflections	21426	17172
R_merge_	8.7 (65.7)	7.6 (54.7)
*I/σI*	27.0 (1.6)	30.7 (1.9)
Completeness (%)	98.9 (93.8)	88.6 (56.1)
Redundancy	6.1 (4.1)	5.7 (4.9)
**Refinement Statistics**		
Resolution (Å)		50.0-2.5
*R*_work_/*R*_free_		23.59/27.56
No. atoms		
Protein		4391
Solvent		27
R.m.s. deviations		
Bond lengths (Å)		0.006
Bond angles (°)		0.838
Isotropic B values (Å^2^)		61.15
Protein		61.27
Water		50.93
Ramachandran		
Favored		98.08
Allowed		1.92
Outliers		0

Values in parentheses correspond to those in the outer resolution shell.

R_merge_ = (|(ΣI−<I>)|)/(ΣI), where <I> is the average intensity of multiple measurements.

R_work_ = Σ_hkl_∥F_obs_(hkl)∥−F_calc_ (hkl)∥/Σ_hkl_|F_obs_(hkl)|.

R_free_ = crossvalidation R factor for 7.3% of the reflections against which the model was not refined.

The C-terminal end of each dimer is splayed open allowing the formation of a helical bundle beginning at residue 875. The SBD^SER^ mutant that facilitates crystallization, positions 884–886, is located within the center of this helical bundle. The alanines at positions 884 and notably 885 are making hydrophobic contacts with the other dimer within the asymmetric unit(Figure 2B). Position 886 is surface exposed and does not appear to play a role in facilitating crystallization. The helix is noticeably kinked after the SBD^SER^ positions suggesting that the substitutions at the SBD^SER^ positions may have altered the overall structure of the SBD to favor the formation of a helical bundle between residues 875–902. To determine whether the SBD^SER^ mutant was affecting protein function, we first tested the oligomeric state of SBD and SBD^SER^ in solution by treating both proteins with the chemical crosslinker glutaraldehyde and resolving the resulting species on SDS-PAGE (Figure 2C). Both SBD and SBD^SER^ readily formed dimers in this assay. We next tested whether Shrm binding was affected by the SBD^SER^ substitution using native gel electrophoresis. Shrm-Rock complexes are more readily visualized using a larger hRock1 fragment, so we utilized amino acids 707–946 of hRock1 in this assay. In this assay, either wildtype or an SBD^SER^ variant of 707–946 was mixed with human Shrm2 SD2 (in the absence of crosslinker), and the proteins resolved by native PAGE. As indicated by the formation of the slower migrating complex, the wildtype and the SER variant exhibit roughly equivalent binding to the SD2, supporting the view that the SBD^SER^ variant of Rock1 is still biochemically functional (Figure 2D). Since SBD^SER^ is dimeric in solution, crystal packing forces, perhaps accentuated by additional hydrophobic interactions found in the SBD^SER^ variant, are likely responsible for the observed helical bundle. This packing arrangement was also observed in an unrelated segment of the Rock1 coiled-coil [56].

### A conserved region within the Rock coiled-coil mediates Shrm binding

**Figure 3:**
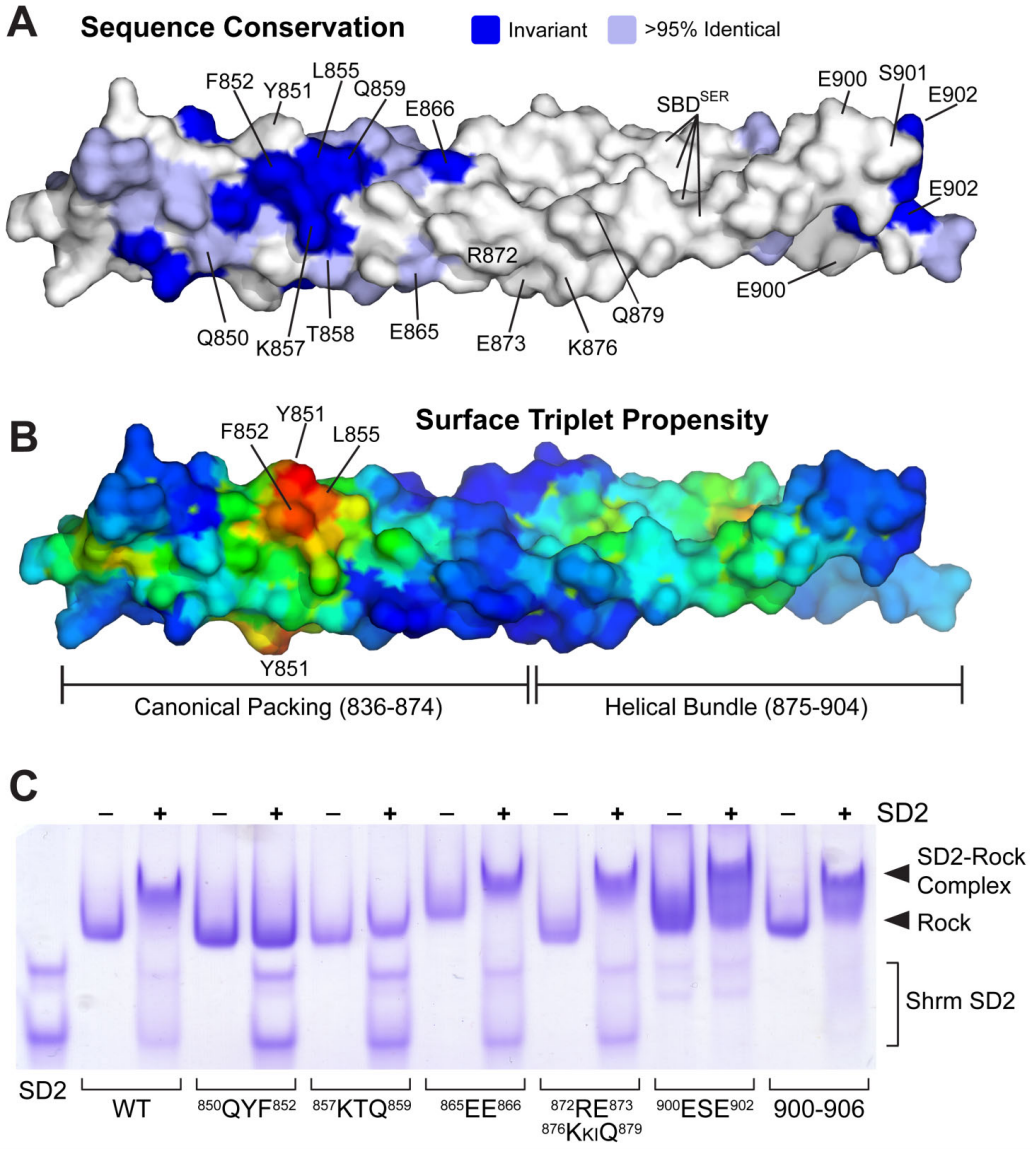
A conserved region on the SBD surface mediates Shrm binding. A) Surface view of the SBD dimer colored by sequence conservation. Residues colored light blue are identical in >95% of the Rock sequences in our alignment, while residues that are invariant across all 14 sequences are color darker blue. Residues that were altered in our mutational analysis are labeled. B) Surface of the SBD dimer colored by Surface Triplet Propensity. Scoring is colored as a heat map with lowest scores in dark blue and the highest scores in red. A prominent patch containing residues Y851 and F852 is indicated. C) Residues within the conserved patch contribute to Shrm binding. Human Shrm2 SD2 was mixed with wild-type Rock1 SBD or the indicated mutant and the formation of a Rock-Shrm complex was detected by native gel electrophoresis.

We used the multiple sequence alignment described earlier (Figure 1A and Figure S1) to map sequence conservation onto the surface of our SBD^SER^ model, reasoning that residues mediating the interaction to the conserved SD2 domain would also be evolutionarily conserved. This analysis revealed the presence of a large conserved stretch of amino acids formed by residues 837 to 866 (Figure S1), that were >95% identical across the sequences we analyzed. These were contained entirely within the canonically packed region, while residues outside of 837–866 contained comparatively fewer conserved residues (Figure 3A). However, given the nature of the coiled-coil, many of these positions may be conserved in order to maintain the coiled-coil dimer or preserve helical propensity. Therefore we scored all atom positions within our model based on their surface triplet propensity [57], an algorithm designed to predict protein-protein and protein-ligand interaction surfaces. Mapping these scores onto the surface of the SBD structure reveals a high scoring patch, formed primarily by residues Y851 and F852 (Figure 3B, red), that is contained within the region of high conservation.

To address whether this patch is important for mediating the Rock-Shrm interaction, we generated a series of mutants throughout the Rock1 SBD (Figure 3A), and tested their ability to form a stable complex with the SD2 by native gel electrophoresis. Since the outer surface is quite extended, we chose to generate amino acid substitutions in clusters of adjacent residues, using knowledge of the structure and the coiled-coil heptad positions to avoid changing residues critical for coiled-coil stability. Expression constructs encoding human Rock1 707–946 with alanine substitutions at the identified positions were generated and the resulting proteins purified. We chose to utilize alanine substitutions in order to preserve helical propensity. All of these SBD variants purify equally well and exhibit similar mobility on a native gel, indicating these alterations do not perturb dimerization or the overall structural integrity of the protein. These SBD variants were then tested for their ability to bind the SD2 domain from human Shrm2 (Figure 3C). In comparison to wildtype protein, amino acids substitutions at residues ^865^EE^866^ and ^872^ RE nl K ki Q ^879^ (in which the underlined residues were changed to alanine), had no effect on complex formation, demonstrating that the central portion of the SBD does not play an important role in Shrm binding. In contrast, two other variants used in this study, ^850^QYF^852^ and ^857^KTQ^859^, formed little to no complex with the SD2, indicating significant defects in Shrm binding. These residues are located within the highly conserved patch near the N-terminus of the SBD and demonstrate that this surface plays a prominent role in Shrm binding. The Rock multiple sequence alignment also indicated a strongly conserved patch of residues at positions 897–906, which was not visualized in its native conformation due to packing forces and disorder after residue 902. To examine the role these residues may play in Shrm binding we generated two additional variants, ^900^ESE^902^ and ^900^ESEQLAR^906^. These variants were slightly impaired, but not deficient, for Shrm binding by native gel electrophoresis (Figure 3C).

**Figure 4:**
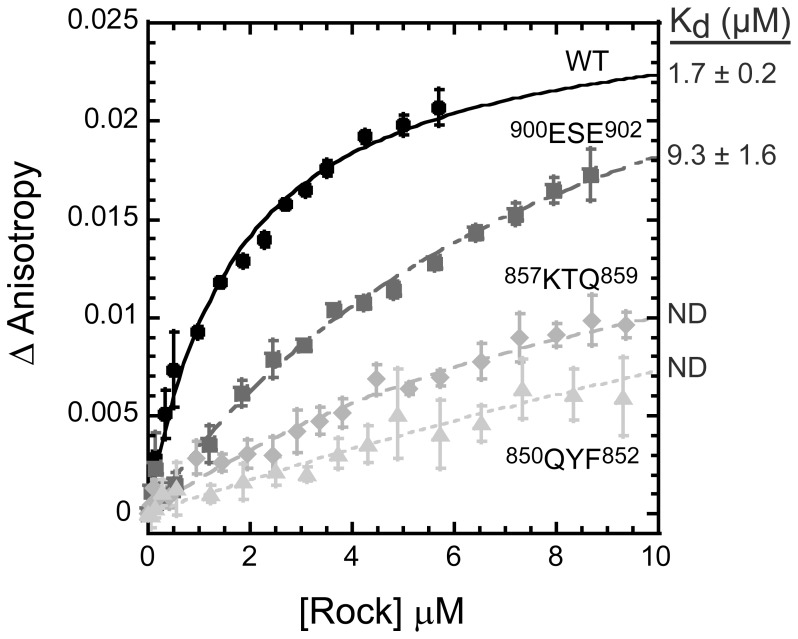
Rock1 SBD variants show decreased interaction with Shrm. Fluorescence anisotropy experiments monitored 50-Green labeled human Shrm2 SD2 domain with increasing concentrations of Rock1 (707–946) containing the indicated amino acid substitutions. The change in anisotropy was fit to Equation 1 to determine binding affinities (*K_d_*) as indicated.

To obtain a quantitative understanding of the effect of these mutants on Shrm binding, we measured the effect of selected Rock1 SBD mutants (^850^QYF^852^, ^857^KTQ^859^, and ^900^ESE^902^) on binding affinity using fluorescence anisotropy as described earlier. Consistent with our analysis by native PAGE, ^900^ESE^902^ had only a modest impact on Shrm binding, resulting in an ∼5.5-fold decrease in binding affinity, while ^850^QYF^852^ and ^857^KTQ^859^ were severely compromised, preventing an accurate measure of binding affinity (Figure 4). While we cannot rule out a direct and important role for residues 900–906, the data presented here are most consistent with the presence of a single binding site for Shrm located between 839–860, with surface exposed residues between 850–859 serving as critical binding determinants.

### Residues important for Shroom-Rock colocalization

**Figure 5:**
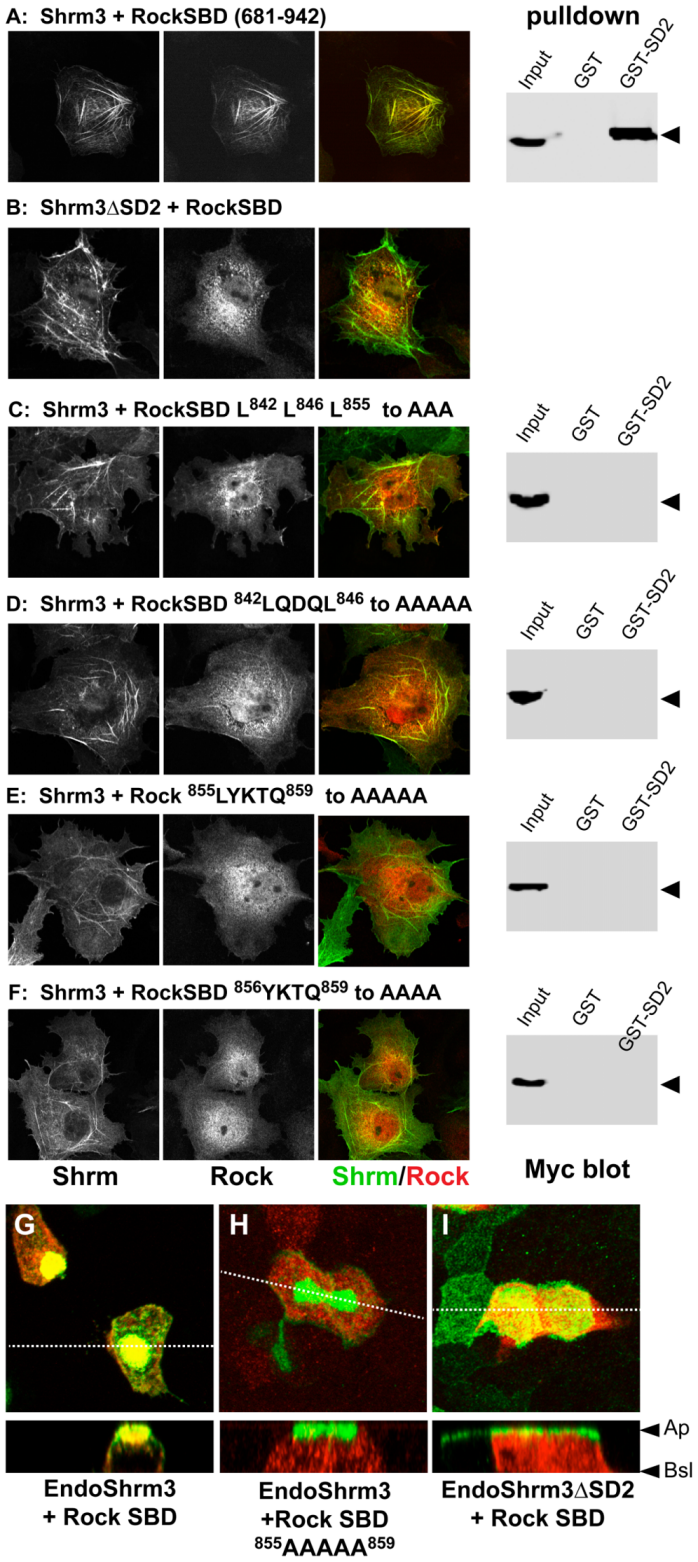
The Rock1 SBD is required for localization with Shrm3. (A–F) Myc-tagged wild type or SBD variants of hRock1 (681–942) were co-expressed with wildtype Shrm3 or a Shrm3 variant lacking the SD2 in Cos7 cells and stained to detect Shrm3 (green) or the myc tag (red). The right-hand panels in A, C–F depict the results of pulldown assays to detect the interaction of the indicated SBD variant and the Shrm3 SD2. Binding of the Rock SBD variants was tested by using immobilized GST-Shrm3 SD2 and lysates from HEK293 cells expressing the indicated SBD protein, followed by western blotting to detect the myc-tagged SBD proteins. Input = total cell lysate, GST = pulldown using GST bound to beads, GST-SD2 = GST-Shrm3-SD2 bound to beads. Arrowhead denotes the myc-tagged Rock protein. (G–I) T23 MDCK epithelial cells were transfected with expression vectors for EndoShrm3 and Rock1 SBD (G), EndoShrm3 and Rock1-SBD ^855^LYKTQ^859^ to ^855^AAAAA^859^ (H) or EndoShrm3ΔSD2 and Rock1-SBD (I), grown on transwell filters overnight to form confluent monolayers, and stained to detect EndoShrm3 (green) and Rock-SBD (red). Dashed lines indicate the position of the Z-projections that are shown in the lower panels. Ap, apical surface; Bsl, basal surface.

To investigate the role of the Shrm-Rock interaction in cellular morphology, we first assessed the ability of Shrm to recruit amino acids 681–942 of Rock1, containing the SBD, to specific subcellular locations in Cos7 cells. For these studies we used the short version of Shrm3 that lacks the N-terminal PDZ domain but contains the actin-binding region and the SD2 domain. This naturally occurring Shrm3 isoform binds both actin and Rock and retains the ability to induce apical constriction in polarized epithelial cells [44], [58]. In these cells, Shrm3 localizes specifically to actin stress fibers and cortical actin (Figure 5A) [44]. When co-expressed with Shrm3, amino acids 681–942 of Rock are efficiently recruited to actin stress fibers. Consistent with previous results [42], recruitment is dependent on the SD2 domain, as a variant of Shrm3 lacking the SD2 (Shrm3ΔSD2) is incapable of recruiting Rock to stress fibers and the Rock protein is diffusely distributed in the cytoplasm (Figure 5B). To verify that the SBD is responsible for the co-localization of Rock with Shrm3, we tested a number of SBD variants for Shrm3-dependent recruitment to actin stress fibers in Cos7 cells. We designed and generated mutations resulting in alanine substitution in highly conserved stretches of amino acids that we predicted would disrupt either the Rock-Shrm interface or perturb Rock coiled-coil interactions. These mutated amino acid segments include L^842^L^846^L^855^, ^842^LQDQL^846^, ^855^LYKTQ^859^, and ^856^YKTQ^859^. All of these Rock variants exhibited reduced recruitment to actin stress fibers by Shrm3, albeit with different severity (Figure 5C–F). The ^855^LYKTQ^859^, and ^856^YKTQ^859^ alanine substitutions virtually eliminated recruitment to stress fibers, indicating this region plays a significant role in the Shrm-Rock interaction (Figure 5E and 5F). Importantly, these data are consistent with in vitro binding experiments described above. In contrast, the ^842^LQDQL^846^ variant exhibits reduced recruitment to stress fibers in cells expressing Shrm3, suggesting that it plays a role in Shrm binding but is not absolutely required (Figure 5D). It is interesting to note that the triple Leucine substitution eliminated binding (Figure 5C). This mutation was generated prior to solving the crystal structure and was based on conservation and the hypothesis that these residues could mediate Rock-Rock or Rock-SD2 binding via coiled-coil or leucine zipper interactions. Combined with the results described above and the crystal structure, we predict that the triple leucine mutant may disrupt both the coiled-coil nature of the rock dimer, mediated by residues L^842^and L^846^ , which are buried, and the binding interface, mediated by L^855^, which is surface exposed. Based on the large binding interface and numerous other coiled-coil interactions, it is unlikely that this mutation completely disrupts the dimer. However, it may cause localized disruption of the dimer that perturbs the structure of the SD2 binding sight. This is supported by our observations that the triple leucine variant is expressed equally well in cells and bacteria and exhibits similar elution profiles during purification.

To verify that the co-localization results were caused by the inability of these Rock variants to interact with Shroom3, we tested their ability to bind Shroom3 using a pulldown assay. To accomplish this, the Rock SBD variants were expressed in 293HEK cells and assayed for the ability to bind purified GST-Shrm3 SD2 immobilized on beads (Figure 5, right panels). Consistent with the co-localization data, only the WT Rock SBD protein is pulled down by GST-Shrm3 SD2, suggesting that the Rock SBD substitution variants are incapable of binding to the SD2.

To ensure that our results were not affected by our choice of cell line and that the same mechanisms are used at the apical surface of polarized epithelial cells, we verified the role of the SBD in Shrm-Rock co-localization in MDCK cells. To accomplish this, we co-expressed either WT Rock 681–942 or the ^855^LYKTQ^859^ to ^855^AAAAA^859^ variant with a previously described fusion protein, EndoShrm3, that consists of the apically targeted transmembrane protein Endolyn and the C-terminus of Shrm3 containing the SD2 [36]. EndoShrm3 is localized to the apical surface of polarized cells and induces dramatic apical constriction. When co-expressed in MDCK cells, we observe strong co-localization of EndoShrm3 and the Rock SBD (Figure 5G) at the apical surface. In contrast, the ^855^LYKTQ^859^ to ^855^AAAAA^859^ variant is cytoplasmic and not recruited to the apical surface with EndoShrm3 (Figure 5H). The SD2 is required for this interaction as a version of EndoShrm3 that lacks the SD2 does not cause apical constriction and does not recruit wildtype SBD to the apical surface (Figure 5I). These data indicate that the SD2-SBD interaction is required for efficient co-localization of Shrm3 and Rock in vivo.

### The SBD is required for Shroom3-induced apical constriction

**Figure 6:**
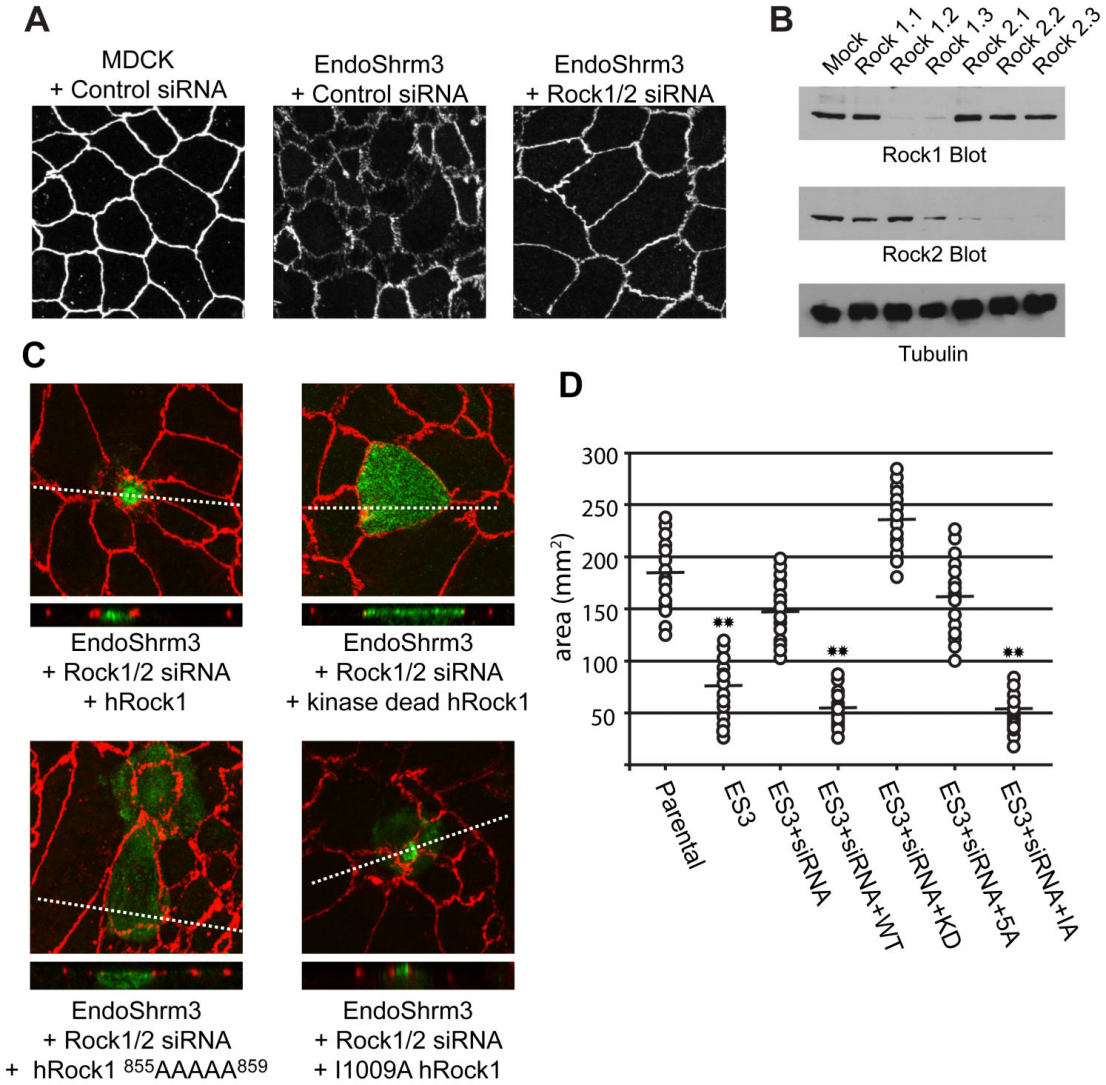
Rock-Shrm interaction is required for apical constriction. (A) Parental or EndoShrm3 expressing T23 MDCK cells were treated with either control or Rock1 and 2 specific siRNAs and stained to detect ZO1. (B) Western blot analysis of Rock1 and Rock 2 knock-down in MDCK cells. (C) T23 MDCK cells expressing EndoShrm3 treated with Rock1/2 siRNA for 2 days, transfected with the indicated hRock1 expression vectors, grown on transwell membranes for 24 hours, and stained to detect ZO1 and hRock1. Z-projections are shown in smaller panels. (D) Quantification of rescue of apical constriction by Rock1 variants. Apical area, as determined by the outline of ZO1 staining, was measured for parental, EndoShrm3 (ES3) expressing, and EndoShrm3 cells treated with Rock1 siRNA (+siRNA). For rescue experiments, apical areas of only those cells that expressed the indicated Rock1 proteins (WT = wildtype, KD = kinase dead; IA = RhoA binding domain mutant, 5A = SBD mutant ^855^LYKTQ^859^ to ^855^AAAAA^859^) were measured. Results are shown for 15 cells picked at random from a single experiment. The horizontal line indicates the average apical area while ** indicates *p* = 0.001 relative to the apical area of parental cells.

The above results indicate that a central coiled-region of Rock is sufficient for Shrm3-mediated subcellular localization and that this activity requires amino acids 855–859 of human Rock1. To investigate the role of this interaction during apical contractility, we have established a knock-down/add-back assay to evaluate the ability of Rock variants to participate in Shrm3-mediated apical constriction. We have previously engineered T23 MDCK cells that inducibly express the EndoShrm3 fusion described above [36]. Upon induction of EndoShrm3 via withdrawal of doxycyclin, cells exhibit robust apical constriction and a marked disruption of tight junction organization as judged by ZO1 staining (Figure 6A). To dissect the mechanism of Rock function in this process, we utilized siRNA-mediated knock-down of canine Rock1 and Rock2. As demonstrated by western blotting, we can successfully deplete Rock1 and Rock2 in MDCK cells (Figure 6B). Knock-down of Rock1 or Rock2 independently is unable to prevent SD2-induced apical constriction (data not shown) while simultaneous knock-down of both Rock1 and Rock2 effectively prevents this phenotype, suggesting that Rock1 and Rock2 are redundant in Shrm-induced apical constriction (Figure 6A). Importantly, apical constriction is rescued following re-expression of human Rock1 in cells that have been treated with siRNA (Figure 6C). The ability of Rock1 to restore apical constriction is dependent on its catalytic activity, as a kinase dead Rock mutant (K105A, KD) cannot restore apical constriction [30]. It should be noted that both wildtype and the kinase dead Rock1 are recruited to the apical surface (Figure 6C). Expression of wildtype Rock1 in uninduced MDCK cells does not cause apical constriction and is not recruited to the apical surface (data not shown). In contrast to wildtype Rock1, the Rock1 variant harboring the ^855^LYKTQ^859^ to ^855^AAAAA^859^ (Rock1-5A) mutation is neither recruited to the apical surface nor able to effectively rescue apical constriction (Figure 6C). To quantify these data, the apical areas of indicated MDCK cell populations were measured using the area enclosed by ZO1 as the readout for apical area (Figure 6D). For the parental, EndoShrm3, and siRNA treated cells, cells were selected at random and measured. For the Rock1 rescue experiments, only those cells that expressed the indicated Rock1 protein were measured. Taken together, these data indicate that the ability of Rock1 to mediate Shrm3 induced apical constriction is dependent on its ability to bind the SD2.

It is currently unclear if the Shrm-Rock interaction is sufficient to activate Rock or if binding to RhoA is also required. To address this, we performed the above experiments with an I1009A (IA) variant of Rock1 that is unable to bind active RhoA [59]. When utilized in this assay, Rock1-IA is recruited to the apical surface and effectively rescues apical constriction (Figure 6C and 6D). This result would suggest that in this context, RhoA binding is not necessary for Rock1 to mediate SD2-induced apical constriction. However, it is possible that the enhanced apical localization and over expression of Rock is sufficient to overcome the need for active RhoA and additional experiments specifically addressing the activation of Rock1 by the SD2 are necessary.

## Discussion

**Figure 7:**
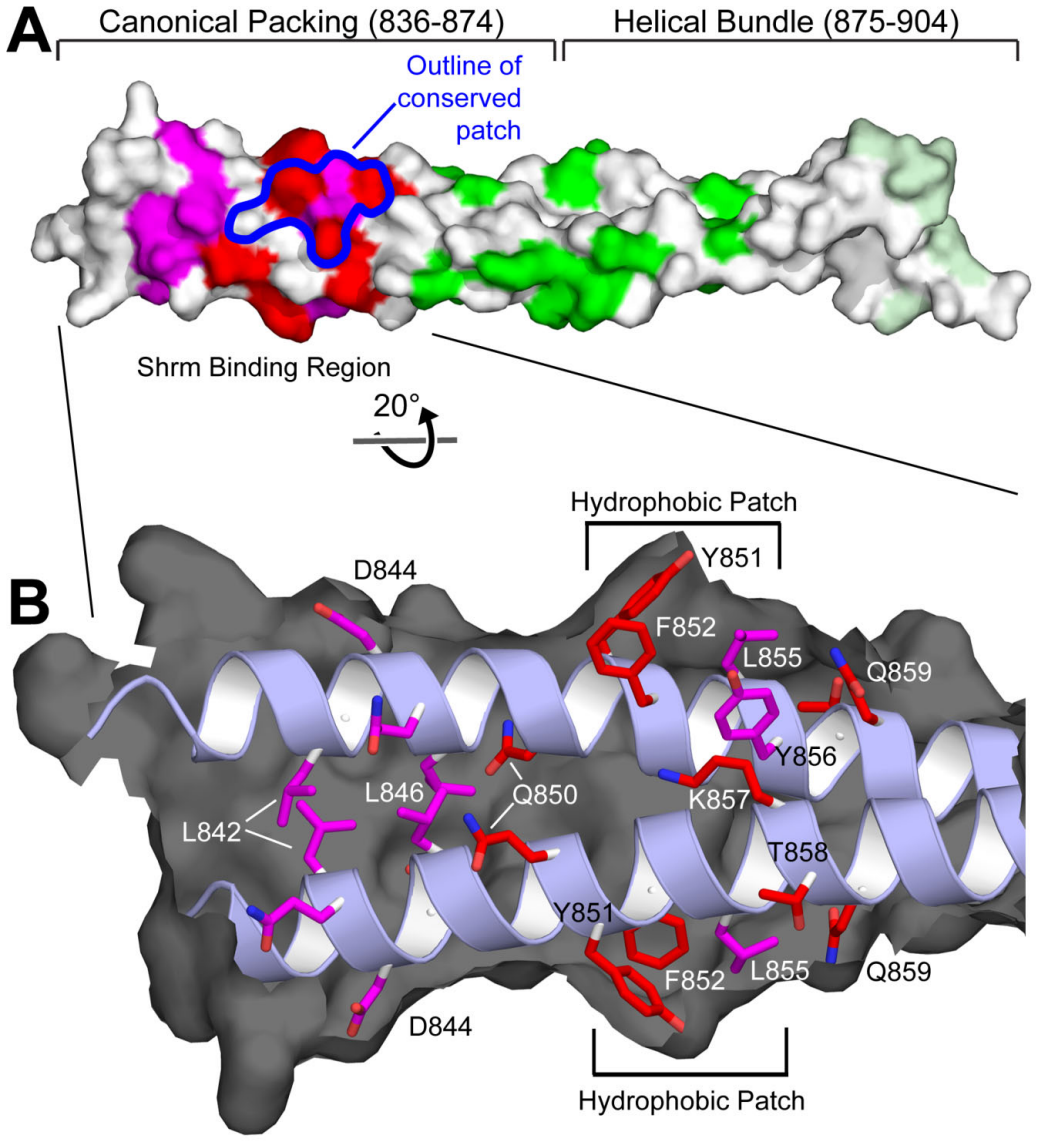
Opposing Shrm binding sites within the SBD. A) Surface representation of the Rock SBD colored by the effect of substitutions in cell based and in vitro assays. Included are positions which altered Shrm colocalization (magenta), positions which affected SD2 binding in vitro (red), residues which did not affect SD2 binding in vitro (green), and residues 900–902 which had a subtle affect on SD2 binding (pale green). B) Cutaway view of the Shrm binding region. Ribbon diagram and positions of side chains with a demonstrated affect (sticks) are colored as above. Black represents the Rock surface which has been cut away to reveal the backbone and side chains underneath. A hydrophobic patch comprised of residues Y851, F852, and L855 is indicated for each binding site.

The Shrm-Rock signaling module is critical for a number of developmental processes and appears to represent a non-canonical mechanism for the localized activation of actomyosin contractility in polarized epithelial cells. To understand the nature of the Shrm-Rock interaction, we have solved the crystal structure of the Shrm Binding Domain of human Rock1. Consistent with other structures of the Rock coiled-coil region, the SBD is a parallel, coiled-coil dimer. Our data show that the interface between Rock and the SD2 motif is conserved and represents a previously unidentified binding surface in the coiled-coil region on Rock. Based on our data, we predict that the Shrm binding activity is encompassed within residues 842–859 of the human Rock 1SBD (Figure 7A). Within this region, we have identified two highly conserved surface patches consisting of ^850^QYF^852^ and ^857^KTQ^859^ that are essential for Shrm binding and likely constitute a portion of the binding interface between Shrm and Rock. Further, all the substitutions that were tested within the conserved sequence patch resulted in a loss of Shrm binding or colocalization, demonstrating that this patch is functionally important (Figure 7A). Due to the nature of the SBD structure, there is a second equivalent conserved binding patch residing on the opposing face of the coiled-coil. An examination of residues in this region indicates that the side chains for Y851, F852, and L855 are packed together to form a hydrophobic patch that protrudes slightly from the rest of the SBD surface (Figure 7B). This is the only significant hydrophobic sequence within the 842–859 region of Rock1 and we speculate that this hydrophobic patch is playing an important role in mediating Rock-Shrm binding. Alternatively, it is possible that the binding surface is more extensive, containing additional residues from both chains of the Rock dimer, as would be predicted from sequence conservation. K859 and Q850 would be attractive candidates for inclusion into an alternative extended binding surface (Figure 7B). Importantly, either possibility results in the formation of two binding sites on opposite faces of the Rock coiled-coil. Currently, the SBD substitutions that we have analyzed cannot distinguish between these possibilities. However, it should be noted that both models are consistent with our previous studies indicating that the SBD-Rock complex is a heterotetramer consisting of a SBD dimer and two SD2 molecules. Residues that are buried within the coiled-coil and mediate strand interactions could abrogate binding in two ways. First, defects in the coiled-coil might perturb the overall structure of the dimer, thus distorting the binding site. Alternatively, if residues from both monomers of Rock1 contribute to the Shrm binding site, mutations that prevent coiled-coil formation within Rock may ablate the binding site altogether.

Our data indicate that both Rock1 and Rock2 can function in Shrm3-mediated apical constriction and changes in cell morphology. This is consistent with previous results showing that the SD2 can bind both Rock1 and Rock2 [37]. Additionally, our in vitro and in vivo approaches have identified residues that are essential for binding and colocalization respectively, and these residues are highly conserved in Rock proteins from most metazoans. Exceptions are Rock proteins from *C. elegans* and sponges, in which the LYKTQ sequence is not conserved. These were not included in our alignment however because they also lack a discernible Shrm homolog. These data suggest that the Shrm-Rock interaction has been maintained across animal evolution and may represent an ancient signaling module that regulates cell behavior during morphogenic events.

Mapping of critical Shrm-binding residues indicates that they are positioned within approximately 50 amino acids of the Rho-binding domain. We have previously shown that the SD2 and active RhoA can likely bind simultaneously to Rock in vitro [10]. However, we show here that Rock proteins lacking the RhoA binding site are still apically recruited by Shrm3 and can mediate apical constriction. Although this is an artificial system, these results would suggest that while Rock can bind both Shrm and RhoA, these binding events could independently regulate Rock function during distinct biological processes. However, there could be instances where inputs from both Shrm and RhoA are required to get a specific degree of Rock activation or localization. These results seem to contradict previous results suggesting that Shroom-mediate apical constriction requires RhoA activity [38]. While these previous studies placed RhoA in the pathway, they did not place it upstream or downstream of Shrm3 or Rock. It possible that RhoA activity is necessary for the assembly and organization of the actin cytoskeleton associated with apical cell-cell adhesions that are required for proper Shrm3 localization. This is supported by the observation that basally localized activated RhoA causes the redistribution of ZO1 and Shrm3 to the basal surface [38]. This would suggest that RhoA is upstream of Shrm3 localization and subsequent apical constriction. This idea is further supported by results showing that N-cadherin genetically interacts with Shrm3 in mice [39]. Solving this issue will require direct measurements of Rock catalytic activity in the presence of various combinations of RhoA and Shrm.

The structural studies presented here indicate that the coiled-coil region of Rock contains a well conserved binding site for Shrm proteins. The fact that the SBD is clearly distinct from other defined binding sites in the coiled-coil region of Rock adds another layer complexity to the function and regulation of Rock during numerous biological processes. Our results suggest that Shrm binding is able to recruit Rock and may be sufficient to activate it in the absence of RhoA binding. The ramifications for this are many fold. First, it provides another pathway by which cells can spatially control the activity of Rock in order to regulate specific changes in cytoskeletal organization. Secondly, this shows that there may be ways to target defined aspects of Rock activity while leaving others untouched. This may allow for more specific molecular dissection of Rock function in vivo or as a way to target Rock activity for the purposes of therapeutic development in the treatment of a variety of human diseases.

## Supporting Information

Figure S1 Diagram of Rock1 variants used in this study. Sequence conservation within the SBD region is indicated through an alignment of 22 Rock sequences. Residues colored blue in the alignment are invariant across the aligned sequences. The location of Rock1 variants generated in this study are indicated above alignment and are colored by the effect of the substitution in the indicated assay.
(TIF)
