# Supplementary figures and images for: Structure of a Highly Conserved Domain of Rock1 Required for Shroom-Mediated Regulation of Cell Morphology
Source: PLoS One. 2013 Dec 9;8(12):e81075. doi: 10.1371/journal.pone.0081075 (PMC3857177; doi:10.1371/journal.pone.0081075)

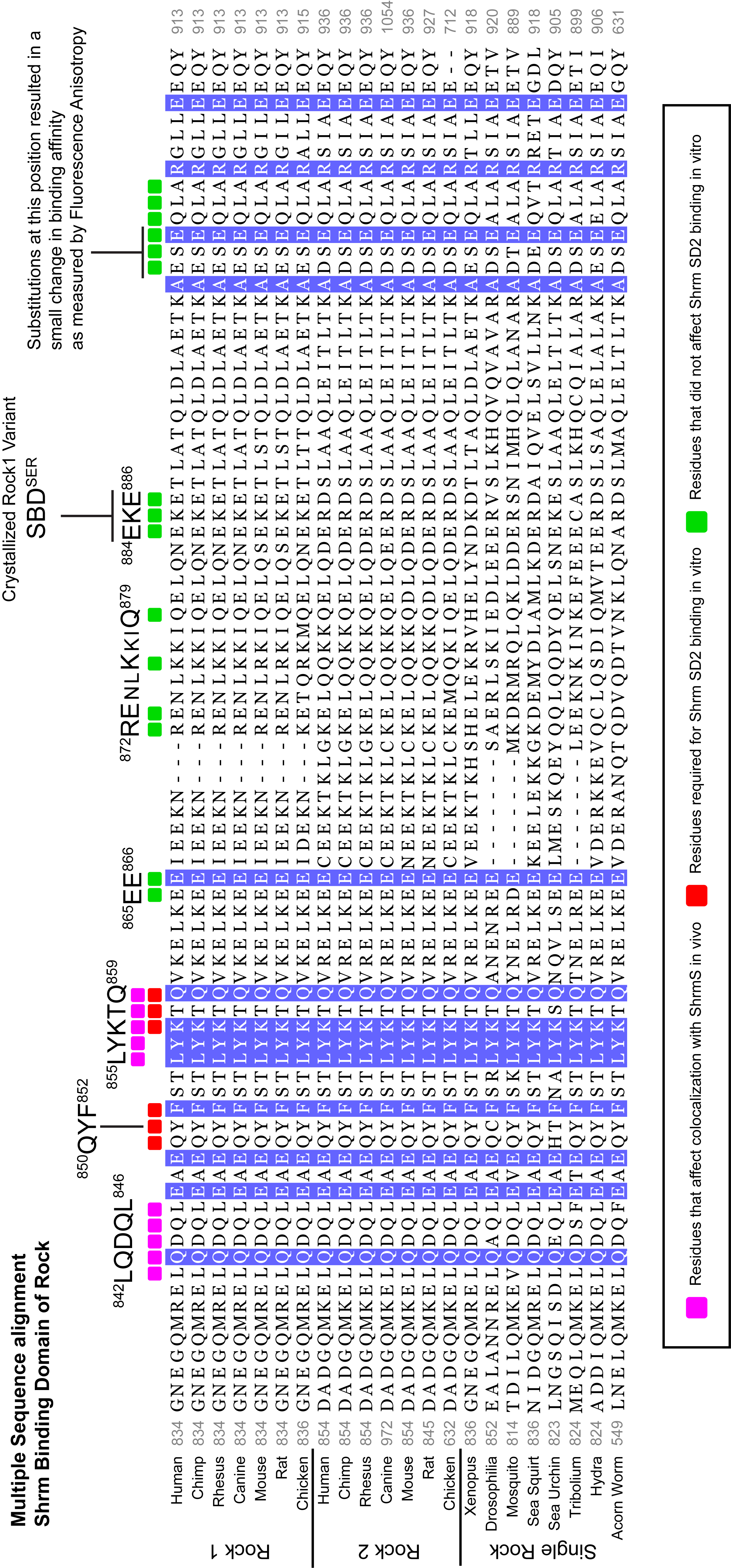

Supplement: Figure S1 — Diagram of Rock1 variants used in this study. Sequence conservation within the SBD region is indicated through an alignment of 22 Rock sequences. Residues colored blue in the alignment are invariant across the aligned sequences. The location of Rock1 variants generated in this study are indicated above alignment and are colored by the effect of the substitution in the indicated assay. (TIF) [file pone.0081075.s001.tif]
